# *catena*-Poly[(*E*)-4,4′-(ethane-1,2-di­yl)dipyridinium [[bis­(thio­cyanato-κ*N*)ferrate(II)]-di-μ-thio­cyanato-κ^2^ *N*:*S*;κ^2^ *S*:*N*]]

**DOI:** 10.1107/S1600536811039924

**Published:** 2011-10-08

**Authors:** Susanne Wöhlert, Inke Jess, Christian Näther

**Affiliations:** a Institut für Anorganische Chemie, Christian-Albrechts-Universität Kiel, Max-Eyth-Strasse 2, 24118 Kiel, Germany

## Abstract

In the crystal structure of the title compound, {(C_12_H_14_N_2_)[Fe(NCS)_4_]}_*n*_, the iron(II) cation is coordinated by four *N*-bonded and two *S*-bonded thio­cyanate anions in a distorted octa­hedral coordination mode. The asymmetric unit consists of half an iron(II) cation and half a protonated (*E*)-4,4′-(ethane-1,2-di­yl)dipyridinium dication, each located on a centre of inversion. In addition, there are two thio­cyanate anions in general positions. The crystal structure consists of Fe—(NCS)_2_—Fe chains in which each iron(II) cation is additionally coordinated by two terminal *N*-bonded thio­cyanate anions. Non-coordinating dipyridinium dications are present between the thiocyanatoferrate(II) chains and are connected to the anions *via* N—H⋯N and N—H⋯S hydrogen-bond interactions.

## Related literature

For coordination polymers based on transition metal thio- and seleno­cyanates, see: Wöhlert *et al.* (2011 ▶); Boeckmann *et al.* (2010 ▶). For a similar structure, see: Wöhlert *et al.* (2010 ▶).
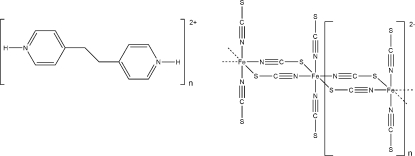


## Experimental

### 

#### Crystal data

(C_12_H_14_N_2_)[Fe(NCS)_4_]
*M* *_r_* = 474.42
Triclinic, 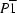

*a* = 5.6818 (3) Å
*b* = 9.0957 (6) Å
*c* = 10.9259 (7) Å
α = 105.586 (5)°
β = 103.633 (5)°
γ = 101.383 (5)°
*V* = 507.65 (5) Å^3^
*Z* = 1
Mo *K*α radiation
μ = 1.17 mm^−1^
*T* = 293 K
0.19 × 0.15 × 0.09 mm

#### Data collection

Stoe IPDS-2 diffractometer
Absorption correction: numerical (*X-SHAPE* and *X-RED32*; Stoe & Cie, 2008) ▶ *T*_min_ = 0.806, *T*_max_ = 0.899
7638 measured reflections
2101 independent reflections
1838 reflections with *I* > 2σ(*I*)
*R*_int_ = 0.030

#### Refinement

*R*[*F* ^2^ > 2σ(*F* ^2^)] = 0.026
*wR*(*F* ^2^) = 0.063
*S* = 1.03
2101 reflections
124 parameters
H-atom parameters constrained
Δρ_max_ = 0.26 e Å^−3^
Δρ_min_ = −0.40 e Å^−3^

### 

Data collection: *X-AREA* (Stoe & Cie, 2008) ▶; cell refinement: *X-AREA* ▶; data reduction: *X-AREA* ▶; program(s) used to solve structure: *SHELXS97* (Sheldrick, 2008 ▶); program(s) used to refine structure: *SHELXL97* (Sheldrick, 2008 ▶); molecular graphics: *XP* in *SHELXTL* (Sheldrick, 2008 ▶) and *DIAMOND* (Brandenburg, 1999 ▶); software used to prepare material for publication: *XCIF* in *SHELXTL*.

## Supplementary Material

Crystal structure: contains datablock(s) I, global. DOI: 10.1107/S1600536811039924/bt5657sup1.cif

Structure factors: contains datablock(s) I. DOI: 10.1107/S1600536811039924/bt5657Isup2.hkl

Additional supplementary materials:  crystallographic information; 3D view; checkCIF report

## Figures and Tables

**Table:** 

Fe1—N1	2.1011 (15)
Fe1—N2	2.1376 (14)
Fe1—S2^i^	2.6729 (5)

**Table:** 

N1^ii^—Fe1—N1	180
N1^ii^—Fe1—N2	88.06 (6)
N1—Fe1—N2	91.94 (6)
N2^ii^—Fe1—S2^iii^	86.73 (4)
N1—Fe1—S2^i^	86.79 (4)

Symmetry codes: (i) 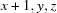; (ii) 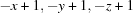; (iii) 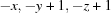.

**Table 2:** Hydrogen-bond geometry (Å, °)

*D*—H⋯*A*	*D*—H	H⋯*A*	*D*⋯*A*	*D*—H⋯*A*
N10—H10*A*⋯N1	0.86	2.34	3.029 (2)	137
N10—H10*A*⋯S2^i^	0.86	2.73	3.4369 (15)	141

Symmetry code: (i) 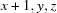.

**Fig. 1.**
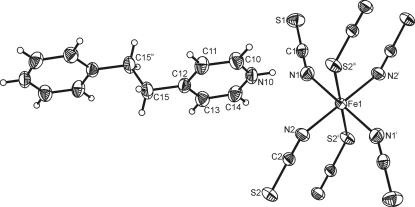
Crystal structure of the title compound with labelling and displacement ellipsoids drawn at the 30% probability level. Symmetry code: i = -x + 1, -y + 1, -z + 1; ii = -x, -y +1, -z +1; iii = x+1, y, z; iv = -x, -y +2, -z.

**Fig. 2.**
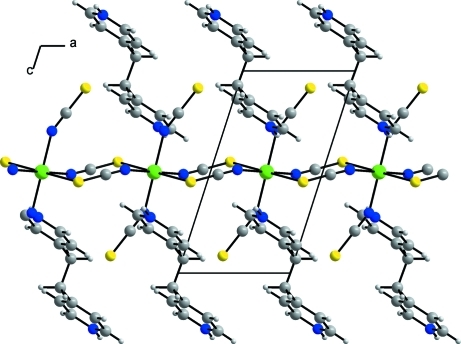
: Crystal structure of the title compound viewed along the crystallographic b axis.

## supplementary crystallographic information

### Comment

In our current work, we are interested in the structure and properties of new coordination polymers based on transition metal thio- and selenocyanates (Wöhlert *et al.*, 2011; Boeckmann, Wriedt & Näther, 2010). In our ongoing investigation in this field we have reacted iron(II) sulfate heptahydrate, potassium thiocyanate and E-1,2-bis(4-pyridyl)-ethane in water. In this reaction red single crystals of the title compound were obtained, which were identified by single crystal X-ray diffraction.

The title compound of composition [Fe(NCS)_4_]_n_-E-1,2-bis(4-pyridinium)-ethane (Fig. 1) represents an 1-D coordination polymer, in which each iron(II) cation is connected by four µ-1,3 bridging thiocyanato anions into chains that elongate in the direction of the crystallographic *a* axis (Fig. 2). Between these chains noncoordinating protonated E-1,2-bis(4-pyridinium)-ethane ligands are found, that are linked to the anions by weak hydrogen bonding interactions (Table 1). The FeN_4_S_2_ octahedron is slightly distorted with two long Fe—SCN distances of 2.6729 (5) Å and short Fe—NCS distances of 2.1011 (15) and 2.1376 (14)Å. The angles arround the metal cations range from 86.73 (5) to 93.27 (4) and 180° (Tab. 1). The shortest intramolecular Fe···Fe distance amounts to 5.6818 (3) Å and the shortest intermolecular Fe···Fe distance amounts to 9.0957 (6) Å. It must be noted that the structure is very similar but not isotypic to that of iron(II) thiocyanate and E-1,2-bis(4-pyridinium)-ethylene reported recently (Wöhlert *et al.*, 2010).

### Experimental

FeSO_4_^.^7H_2_O and 1,2-bis(4-pyridyl)-ethane were obtained from Sigma Aldrich. KNCS are obtained from Alfa Aesar. 0.6 mmol (168.0 mg) FeSO_4_^.^7H_2_O, 1.2 mmol (117.7 mg) KNCS and 0.15 mmol (27.2 mg) 1,2-bis(4-pyridyl)-ethane were reacted with 1 mL H_2_O in closed test-tube at 120°C for three days. On cooling red block-shaped single crystals of the title compound were obtained in a mixture with a second crystalline phase that was not yet identified.

### Refinement

All H atoms were located in difference map but were positioned with idealized geometry and were refined using a riding model with *U*_eq_(H) = 1.2 *U*_eq_(C,N)   with C—H = 0.93 Å and N—H = 0.86 Å.

### Crystal data

**Table:**

(C_12_H_14_N_2_)[Fe(NCS)_4_]	*Z* = 1
*M**_r_* = 474.42	*F*(000) = 242
Triclinic, *P*1	*D*_x_ = 1.552 Mg m^−^^3^
Hall symbol: -P 1	Mo *K*α radiation, λ = 0.71073 Å
*a* = 5.6818 (3) Å	Cell parameters from 7638 reflections
*b* = 9.0957 (6) Å	θ = 2.0–26.5°
*c* = 10.9259 (7) Å	µ = 1.17 mm^−^^1^
α = 105.586 (5)°	*T* = 293 K
β = 103.633 (5)°	Block, red
γ = 101.383 (5)°	0.19 × 0.15 × 0.09 mm
*V* = 507.65 (5) Å^3^	

### Data collection

**Table:**

Stoe IPDS-2 diffractometer	2101 independent reflections
Radiation source: fine-focus sealed tube	1838 reflections with *I* > 2σ(*I*)
graphite	*R*_int_ = 0.030
ω scans	θ_max_ = 26.5°, θ_min_ = 2.0°
Absorption correction: numerical (*X-SHAPE* and *X-RED32*; Stoe & Cie, 2008)	*h* = −7→6
*T*_min_ = 0.806, *T*_max_ = 0.899	*k* = −11→11
7638 measured reflections	*l* = −13→13

### Refinement

**Table:**

Refinement on *F*^2^	Primary atom site location: structure-invariant direct methods
Least-squares matrix: full	Secondary atom site location: difference Fourier map
*R*[*F*^2^ > 2σ(*F*^2^)] = 0.026	Hydrogen site location: inferred from neighbouring sites
*wR*(*F*^2^) = 0.063	H-atom parameters constrained
*S* = 1.03	*w* = 1/[σ^2^(*F*_o_^2^) + (0.0393*P*)^2^ + 0.0464*P*] where *P* = (*F*_o_^2^ + 2*F*_c_^2^)/3
2101 reflections	(Δ/σ)_max_ < 0.001
124 parameters	Δρ_max_ = 0.26 e Å^−^^3^
0 restraints	Δρ_min_ = −0.40 e Å^−^^3^

### Special details

**Table:**

Geometry. All esds (except the esd in the dihedral angle between two l.s. planes) are estimated using the full covariance matrix. The cell esds are taken into account individually in the estimation of esds in distances, angles and torsion angles; correlations between esds in cell parameters are only used when they are defined by crystal symmetry. An approximate (isotropic) treatment of cell esds is used for estimating esds involving l.s. planes.
Refinement. Refinement of F^2^ against ALL reflections. The weighted R-factor wR and goodness of fit S are based on F^2^, conventional R-factors R are based on F, with F set to zero for negative F^2^. The threshold expression of F^2^ > 2sigma(F^2^) is used only for calculating R-factors(gt) etc. and is not relevant to the choice of reflections for refinement. R-factors based on F^2^ are statistically about twice as large as those based on F, and R- factors based on ALL data will be even larger.

### Fractional atomic coordinates and isotropic or equivalent isotropic displacement parameters (Å^2^)

**Table:**

	*x*	*y*	*z*	*U*_iso_*/*U*_eq_	
Fe1	0.5000	0.5000	0.5000	0.02833 (10)	
N1	0.4723 (3)	0.45516 (18)	0.29744 (15)	0.0398 (3)	
C1	0.5464 (3)	0.40682 (18)	0.20845 (16)	0.0311 (3)	
S1	0.64953 (10)	0.33771 (7)	0.08533 (5)	0.04980 (14)	
N2	0.2339 (3)	0.63622 (17)	0.48892 (15)	0.0356 (3)	
C2	0.0753 (3)	0.69028 (18)	0.51257 (15)	0.0283 (3)	
S2	−0.14718 (8)	0.76903 (5)	0.54918 (5)	0.03592 (12)	
N10	0.4056 (3)	0.77059 (17)	0.27628 (15)	0.0384 (3)	
H10A	0.4891	0.7200	0.3178	0.046*	
C10	0.5213 (3)	0.9178 (2)	0.28629 (19)	0.0413 (4)	
H10	0.6894	0.9642	0.3374	0.050*	
C11	0.3919 (4)	1.0005 (2)	0.22104 (18)	0.0399 (4)	
H11	0.4724	1.1029	0.2273	0.048*	
C12	0.1399 (3)	0.9314 (2)	0.14537 (16)	0.0333 (4)	
C13	0.0289 (3)	0.7782 (2)	0.13838 (18)	0.0390 (4)	
H13	−0.1393	0.7289	0.0886	0.047*	
C14	0.1652 (4)	0.6990 (2)	0.20418 (19)	0.0410 (4)	
H14	0.0905	0.5958	0.1986	0.049*	
C15	−0.0056 (4)	1.0190 (2)	0.07142 (17)	0.0416 (4)	
H15A	0.0637	1.1324	0.1177	0.050*	
H15B	−0.1800	0.9896	0.0701	0.050*	

### Atomic displacement parameters (Å^2^)

**Table:**

	*U*^11^	*U*^22^	*U*^33^	*U*^12^	*U*^13^	*U*^23^
Fe1	0.02747 (17)	0.03565 (18)	0.03103 (17)	0.01680 (13)	0.01363 (13)	0.01564 (13)
N1	0.0459 (9)	0.0434 (8)	0.0355 (8)	0.0179 (7)	0.0150 (7)	0.0153 (6)
C1	0.0323 (8)	0.0272 (7)	0.0324 (8)	0.0078 (6)	0.0059 (7)	0.0116 (6)
S1	0.0492 (3)	0.0540 (3)	0.0424 (3)	0.0118 (2)	0.0221 (2)	0.0048 (2)
N2	0.0292 (7)	0.0391 (7)	0.0465 (8)	0.0149 (6)	0.0158 (6)	0.0192 (6)
C2	0.0246 (7)	0.0288 (7)	0.0327 (8)	0.0073 (6)	0.0078 (6)	0.0130 (6)
S2	0.0281 (2)	0.0341 (2)	0.0472 (2)	0.01455 (16)	0.01351 (18)	0.01003 (18)
N10	0.0410 (8)	0.0391 (8)	0.0416 (8)	0.0172 (6)	0.0088 (7)	0.0226 (6)
C10	0.0339 (9)	0.0418 (9)	0.0442 (10)	0.0083 (7)	0.0028 (8)	0.0173 (8)
C11	0.0460 (10)	0.0306 (8)	0.0422 (9)	0.0093 (7)	0.0082 (8)	0.0161 (7)
C12	0.0414 (9)	0.0376 (8)	0.0270 (7)	0.0192 (7)	0.0115 (7)	0.0135 (7)
C13	0.0336 (9)	0.0424 (9)	0.0391 (9)	0.0082 (7)	0.0061 (7)	0.0165 (8)
C14	0.0449 (10)	0.0343 (9)	0.0450 (10)	0.0072 (7)	0.0115 (8)	0.0198 (8)
C15	0.0517 (11)	0.0469 (10)	0.0369 (9)	0.0292 (9)	0.0128 (8)	0.0204 (8)

### Geometric parameters (Å, °)

**Table:**

Fe1—N1^i^	2.1011 (15)	N10—H10A	0.8600
Fe1—N1	2.1011 (15)	C10—C11	1.369 (2)
Fe1—N2^i^	2.1376 (14)	C10—H10	0.9300
Fe1—N2	2.1376 (14)	C11—C12	1.391 (3)
Fe1—S2^ii^	2.6729 (5)	C11—H11	0.9300
Fe1—S2^iii^	2.6729 (5)	C12—C13	1.385 (2)
N1—C1	1.163 (2)	C12—C15	1.504 (2)
C1—S1	1.6157 (18)	C13—C14	1.368 (3)
N2—C2	1.156 (2)	C13—H13	0.9300
C2—S2	1.6472 (16)	C14—H14	0.9300
S2—Fe1^iv^	2.6729 (5)	C15—C15^v^	1.525 (4)
N10—C14	1.333 (2)	C15—H15A	0.9700
N10—C10	1.333 (2)	C15—H15B	0.9700
			
N1^i^—Fe1—N1	180.000 (1)	C10—N10—H10A	118.8
N1^i^—Fe1—N2^i^	91.94 (6)	N10—C10—C11	119.77 (16)
N1—Fe1—N2^i^	88.06 (6)	N10—C10—H10	120.1
N1^i^—Fe1—N2	88.06 (6)	C11—C10—H10	120.1
N1—Fe1—N2	91.94 (6)	C10—C11—C12	120.07 (16)
N2^i^—Fe1—N2	180.000 (1)	C10—C11—H11	120.0
N1^i^—Fe1—S2^ii^	86.79 (4)	C12—C11—H11	120.0
N1—Fe1—S2^ii^	93.21 (4)	C13—C12—C11	117.78 (15)
N2^i^—Fe1—S2^ii^	86.73 (4)	C13—C12—C15	121.12 (16)
N2—Fe1—S2^ii^	93.27 (4)	C11—C12—C15	121.09 (16)
N1^i^—Fe1—S2^iii^	93.21 (4)	C14—C13—C12	120.42 (16)
N1—Fe1—S2^iii^	86.79 (4)	C14—C13—H13	119.8
N2^i^—Fe1—S2^iii^	93.27 (4)	C12—C13—H13	119.8
N2—Fe1—S2^iii^	86.73 (4)	N10—C14—C13	119.63 (16)
S2^ii^—Fe1—S2^iii^	180.000 (13)	N10—C14—H14	120.2
C1—N1—Fe1	149.29 (13)	C13—C14—H14	120.2
N1—C1—S1	179.28 (15)	C12—C15—C15^v^	111.35 (18)
C2—N2—Fe1	158.76 (13)	C12—C15—H15A	109.4
N2—C2—S2	178.92 (16)	C15^v^—C15—H15A	109.4
C2—S2—Fe1^iv^	98.29 (6)	C12—C15—H15B	109.4
C14—N10—C10	122.34 (15)	C15^v^—C15—H15B	109.4
C14—N10—H10A	118.8	H15A—C15—H15B	108.0

Symmetry codes: (i) −*x*+1, −*y*+1, −*z*+1; (ii) −*x*, −*y*+1, −*z*+1; (iii) *x*+1, *y*, *z*; (iv) *x*−1, *y*, *z*; (v) −*x*, −*y*+2, −*z*.

### Hydrogen-bond geometry (Å, °)

**Table:**

*D*—H···*A*	*D*—H	H···*A*	*D*···*A*	*D*—H···*A*
N10—H10A···N1	0.86	2.34	3.029 (2)	137.
N10—H10A···S2^iii^	0.86	2.73	3.4369 (15)	141.

Symmetry codes: (iii) *x*+1, *y*, *z*.
